# New advances of the androgen receptor in prostate cancer: report from the 1st International Androgen Receptor Symposium

**DOI:** 10.1186/s12967-024-04878-5

**Published:** 2024-01-18

**Authors:** Sherif Mehralivand, Christian Thomas, Martin Puhr, Frank Claessens, Arjanneke F. van de Merbel, Anna Dubrovska, Guido Jenster, Christof Bernemann, Ulrich Sommer, Holger H. H. Erb

**Affiliations:** 1 Department of Urology, Faculty of Medicine, University Hospital Carl Gustav Carus, Technische Universität Dresden, Fetscherstraße 74, 01307 Dresden, Germany; 2 Department of Urology, Medical University of Innsbruck, Innsbruck, Austria; 3 Molecular Endocrinology Laboratory, Department of Cellular and Molecular Medicine, KU Leuven, Leuven, Belgium; 4 Department of Urology, Leiden University Medical Center, Leiden, The Netherlands; 5 OncoRay-National Center for Radiation Research in Oncology, Faculty of Medicine and University Hospital Carl Gustav Carus, Technische Universität Dresden and Helmholtz-Zentrum Dresden-Rossendorf, Dresden, Germany; 6 Institute of Radiooncology-OncoRay, Helmholtz-Zentrum Dresden-Rossendorf, Dresden, Germany; 7 National Center for Tumor Diseases (NCT), Dresden, Germany; 8 German Cancer Research Center (DKFZ), Heidelberg, Germany; 9 Faculty of Medicine, University Hospital Carl Gustav Carus, Technische Universität Dresden, Dresden, Germany; 10 Helmholtz-Zentrum Dresden-Rossendorf (HZDR), Dresden, Germany; 11 German Cancer Consortium (DKTK), Partner Site Dresden and German Cancer Research Center (DKFZ), Heidelberg, Germany; 12 Department of Urology, Erasmus MC Cancer Institute, University Medical Center, Rotterdam, The Netherlands; 13 Department of Urology, University Hospital Muenster, Muenster, Germany; 14 Institut für Pathologie, Medical Faculty, University Hospital Carl Gustav Carus, Technische Universität Dresden, Dresden, Germany

**Keywords:** Androgen receptor, PCa, NR3C4, Androgen deprivation therapy

## Abstract

The androgen receptor (AR) is a crucial player in various aspects of male reproduction and has been associated with the development and progression of prostate cancer (PCa). Therefore, the protein is the linchpin of current PCa therapies. Despite great research efforts, the AR signaling pathway has still not been deciphered, and the emergence of resistance is still the biggest problem in PCa treatment. To discuss the latest developments in AR research, the “1st International Androgen Receptor Symposium” offered a forum for the exchange of clinical and scientific innovations around the role of the AR in prostate cancer (PCa) and to stimulate new collaborative interactions among leading scientists from basic, translational, and clinical research. The symposium included three sessions covering preclinical studies, prognostic and diagnostic biomarkers, and ongoing prostate cancer clinical trials. In addition, a panel discussion about the future direction of androgen deprivation therapy and anti-AR therapy in PCa was conducted. Therefore, the newest insights and developments in therapeutic strategies and biomarkers are discussed in this report.

## Introduction

The androgen receptor (AR) belongs to the nuclear receptor superfamily, has a molecular weight of 110–114 kDa, consists of 910–919 amino acids, and is encoded on the X chromosome (Xq11.2-q12) with 8 exons [1–3]. In line with other representatives of the nuclear receptors superfamily, the AR consists of an amino(N)-terminal transactivation domain, a DNA-binding domain, and a hinge domain which connects the DNA-binding domain with the carboxy-terminal ligand-binding domain (LBD) [4, 5]. Androgens, which include dehydroepiandrosterone, androstenedione, androstenediol, androsterone, testosterone, and dihydrotestosterone, serve as ligands for the AR [6–8]. The AR is expressed in diverse tissues such as the bone, brain, muscle, endometrium, testis, and prostate [9–11]. Therefore, it maintains and differentiates between the reproductive, immune, cardiovascular, neural, and hematopoietic systems. Due to its various functions in the human body, it is unsurprising that it is involved in various disease patterns [12, 13]. These diseases include, among others, psychiatric disorders, spinal-bulbar muscular atrophy, hypogonadism, androgen insensitivity syndrome, benign prostatic hyperplasia, and prostate cancer (PCa). Therefore, AR has been the focus of extensive research for several years.

The “International Androgen Receptor Symposium” provides experts in the AR field with the opportunity to exchange the latest scientific advances and develop new research ideas. The symposium occurred on June 16–17, 2023, at the University Hospital Carl Gustav Carus in Dresden, Germany. This year’s symposium focused on the involvement of the AR in PCa, the second leading cause of cancer-related deaths in men [14].

The development of both prostate and prostate cancer (PCa) is strictly dependent on androgens, and both need a continuous androgenic stimulus to maintain cell growth and function [15–18]. Therefore, the AR influences differentiation, cell proliferation, apoptosis, metabolism, and DNA repair mechanisms in the prostate and PCa [19–22]. As long as the tumor is still localized, treatment with curative intent in the form of radiotherapy (RT) and radical prostatectomy is used [23]. However, if the tumor has already metastasized, only pharmacological therapy with palliative intent is available [24]. Due to the strong dependence of PCa on the androgen-AR signaling axis, this plays a central role in drug-based PCa therapy. Therefore, AR activity is reduced by androgen withdrawal using androgen deprivation therapy (ADT) or targeting the AR directly using anti-androgens. These treatments result in cell cycle arrest and apoptosis induction of the PCa cells and reduce tumor volume [25, 26]. Despite the initial success of ADT and anti-androgen therapy, treatments are only effective for a certain period before disease progression and drug resistance occur [27, 28]. Consequently, there is an urgent clinical need to develop novel therapeutic strategies.

## Session 1: new advances from androgen deprivation therapy

### Current treatment landscape in metastatic prostate cancer

As AR is a pivotal point in PCa therapy, Prof. Thomas reported in his presentation “Current treatment landscape in metastatic prostate cancer” on the current development of PCa treatment and how combination treatment will be implemented.

Until the introduction of docetaxel and novel hormonal agents (e.g., enzalutamide, apalutamide, darolutamide, and abiraterone) in combination with ADT, vintage combination therapy (e.g., bicalutamide + ADT and flutamide + ADT) has only shown a negligible survival benefit [29, 30]. However, since 2014, an era of modern combination treatment has started with an improvement in overall survival (OS) of 19–39%. Based on these results, Prof. Thomas highlighted the current combination options for treating metastasized hormone-sensitive PCa (mHSPC), which has been approved since 2017 [31]. Furthermore, he critically discusses the PEACE-1 and ARASENS triple therapy studies, as various meta-analyses have shown no benefit compared to modern dual combination therapy [32–36].

Next to the advances in mHSPC therapy, Prof. Thomas reported the recent development of PARP inhibitor therapy for metastatic castration-resistant PCa (mCRPC). Since 2020, monotherapy with PARP inhibitors has been challenging for patient treatment, as nationwide homologous recombination repair (HRR) gene mutation screening has been insufficiently established in daily clinical practice. As BRCA1/2 mutations occur in less than 10% of PCa patients, the benefit of ADT combined with PARP inhibition, as suggested by the PROFOUND study, is only beneficial for a small sample size of mCRPC patients [37, 38]. The PROpel study demonstrated that indirect inhibition of the DNA repair system by CYP17A1 inhibition combined with PARP inhibitors is successful, even in patients without HRR mutations. However, patients with HRR mutations still have the greatest benefits. Therefore, reliable large-scale HRR mutation screening for PCa patients seems essential.

At the end of his talk, Prof. Thomas presented the latest TheraP and Vision trial data, suggesting PSMA-radio ligand therapy as the new third-line standard and how the therapy can be implemented into the therapy landscape [39, 40].

### Excursus: application of deep learning in prostate cancer diagnostics

Since the introduction of multiparametric prostate magnetic resonance imaging (MRI), the detection of high-risk PCa has increased by 30% and significantly improved PCa diagnosis [41]. However, the main issue with imaging is the variability between evaluations, as standardization is not feasible. Applying artificial intelligence (AI) to medical imaging can reduce this variability. In his talk, Dr. Sherif Mehralivand presented opportunities for AI in multiparametric prostate MRI evaluations. He introduced the audience to the variability in MRI diagnostics caused by patient population, study design, image acquisition, image interpretation, image processing, and reader experience/training. His studies explain the pitfalls of AI evaluation, such as false-negative and false-positive results. In his investigations, the AI algorithms had a sensitivity from 0.63 to 0.73, comparable with human detection rates [42]. He expects that AI can be used to assist MRI evaluation and that there will be future studies to compare with human performances and prospective multi-reader studies.

## Session 2: new advances from basic and translational research

### The androgen receptor-glucocorticoid receptor axis, an underestimated factor for prostate cancer treatment and source for therapy failure

As the first speaker in the session, Dr. Puhr presented in his talk “The AR-Glucocorticoid Receptor Axis, an underestimated factor for PCa treatment and source for therapy failure”, including his latest findings about the AR-glucocorticoid receptor (GR) axis and its role in AR targeted therapy resistance. Several studies have demonstrated that PCa cells can bypass and survive AR blockade, RT, and chemotherapy treatment due to up-regulation of GR expression and elevated GR signaling [43–46]. Dr. Puhr showed that GR levels were elevated during ADT in used preclinical models and patients. In preclinical investigations, GR knockdown or pharmacological inhibition reduced cell proliferation and tumor growth in multiple preclinical models. However, it was discussed that a first phase I/II trial using the unspecific GR-inhibitor mifepristone combined with the anti-androgen enzalutamide had no significant superior effect in a clinical setting. Therefore, the audience agreed that developing more specific and potent GR inhibitors is highly relevant. In this context, the first results of a phase 1 trial (NCT03674814) with enzalutamide and relacorilant, a specific GR inhibitor, showed that the combination was well tolerated in mCRPC patients [47]. Moreover, Dr. Puhr suggested that the GR downstream target monoamine oxidase A (MAO-A) is a possible new promising target as he revealed an increase during PCa progression. The first clinical trials demonstrated promising results in patients with recurrent PCa [48, 49]. In conclusion, Dr. Puhr postulated that combined AR and GR targeting might be a feasible clinical approach for an optimized, personalized PCa therapy in the future.

### The androgen receptor: it takes two, doesn’t it?

As a second speaker, Prof. Claessens discussed the importance of AR dimerization in AR function and how this process could be exploited therapeutically. In his talk “The AR: it takes two, doesn’t it?” he introduced the audience to the different homodimerization and interaction possibilities of AR. The first dimerization mutant disrupts the so-called N/C interaction, which has been demonstrated in many studies to affect coregulatory binding in vitro. Surprisingly, this did not affect the AR function in a mutant mouse model. This finding challenges the in vivo role of this AR-specific dimerization mode but does not exclude its role in prostate cancer [50]. In contrast, disrupting the LBD dimerization of the AR by a point mutation in the mouse genome leads to androgen insensitivity in the absence of accessory sex glands despite much higher circulating testosterone levels. This phenotype is accompanied by a loss of AR binding to its specific genomic binding sites despite conserved ligand and DNA binding, nuclear translocation, and chromatin binding [51]. The LBD dimerization was then exploited to develop a novel AR inhibitor class called AR DIMs. These inhibitors do not bind to the ligand-binding pocket, similar to known AR antagonists, but to the dimerization surface. They inhibit LBD dimerization and reduce AR transactivation as well as the proliferation of AR-positive PCa cell lines [52]. They even affect the cooperation between AR and its variants and hence could become applicable in castration-resistant PCa.

### The interplay between cancer stem cell markers and androgen receptors in the regulation of cancer metastases and radioresistance

RT is a primary treatment option for localized PCa and regionally unresectable advanced PCa, with curative intent [23, 53]. However, up to 40% of PCa patients still experience recurrence within 10 years after RT, for which there is no widely agreed-upon approach for optimal management [54]. Several studies have revealed that a small population of primitive stem-like PCa cells (cancer stem cells; CSC) within the tumor have a greater resilience to RT than most cells and are directly responsible for tumor recurrence [43, 55–59]. In her talk “The interplay between CSC markers and AR in the regulation of cancer metastases and radioresistance,” Prof. Dubrovska explains why some CSC populations are resistant to therapeutic pressure such as AR deprivation therapy and how they are dynamically regulated by AR signaling [27, 57, 59, 60]. Moreover, because of the elevated expression of some essential DNA repair proteins and activation of pro-survival pathways in CSC, they are also difficult to target by RT [56–58]. Prof. Dubrovska illustrated her latest findings in further detail, explaining the interplay between CSC-related pathways and AR transcriptional programs and how these mechanisms can target metastatic and therapy-resistant CSC.

### Excursus: patient-derived tumor models for personalized therapeutics in prostate cancer

In the session’s final talk, Dr. van de Merbel gave an overview of “Patient-derived tumor models for personalized therapeutics in prostate cancer.” Using her data on the oncolytic virus jin-3 reovirus, she described the advantages and pitfalls of PCa models such as syngeneic mice models, 3D prostate organoids, ex vivo slices, and patient-derived xenografts [61, 62]. She revealed that oncolytic viruses are powerful in stimulating antitumor immune responses and may potentiate immunotherapy. However, Dr. van de Merbel reminded the audience that clinically relevant models represent a significant challenge in PCa, and each preclinical model has intrinsic advantages and restrictions. Therefore, depending on the question, the advantages and disadvantages of each model should be carefully weighed and chosen.

## Session 3: the androgen receptor as a diagnostic and prognostic biomarker

### The androgen receptor: fun artificial and detrimental natural variants

The AR splice variants (AR-V), such as AR-V7 and AR-V3, play an essential role in PCa progression and therapy resistance [63]. Moreover, the AR-V7 variant has been discussed as a biomarker for treatment resistance and poor disease outcome [64–66]. In his talk “The AR: Fun Artificial and Detrimental Natural Variants,” Prof. Jenster introduced the audience to AR-V. He reported his first discoveries in C-truncated AR-V and his deduction at the time that these variants were caused by stop-codon mutations [2]. Moreover, he reported on different artificial AR-co-modulator fusion proteins, which are highly active or dominant-negative, and how these fusion proteins helped to understand the different AR domains and comodulators in AR transactivation [67]. These artificial AR-V show characteristics of variants, such as constitutive activity and promiscuity, found in PCa. Based on this, he wondered whether there are other natural AR-V with characteristics that have been observed with artificial AR constructs, such as hyperactivity, AR-coactivator characteristics, or dominant negative AR-V. Moreover, he believes that based on splicing events happening in the AR gene, we have still not identified all AR-V, and novel ones will appear.

### The potential of the androgen receptor splice variant’s status as a predictive and prognostic biomarker

Following Prof. Jenster’s introduction of variants, Dr. Bernemann continued the topic and reported on the diagnostic and prognostic value of AR-V in PCa. In the first part of his talk, Dr. Bernemann reminded the audience about the fact that despite the initial reports of Antoanarakis that patients with AR-V7 do not respond to AR signaling inhibitors (ASI), further studies reported 19–40% of AR-V7 positive patients responding to ASI [66, 68–70]. However, in his opinion, this issue has not been well discussed in the literature, and more investigations on AR-V7 must be performed. Based on this, Bernemann et al. investigated the potential of AR-V panels, including AR-V3, AR-V7, and AR-V9, as diagnostic or prognostic biomarkers. Based on his data, AR-V are up-regulated by increased AR gene activation in PCa and non-prostate-related fibroblasts. Clinically, the group around Bernemann could not validate the panel as a predictive marker. However, he demonstrated that circulating tumor cells alone have a higher prognostic potential as they include the high PCa heterogeneity. Therefore, he believes the AR-V mRNA does not predict therapy resistance. Prof. Jenster added that, in his experience, the full-length AR mRNA in extracellular vesicles is a much more sensitive predictor of ASI treatment than the AR-V [71].

### Excursus: pathology, biomarkers, digitization, and the prostate – where are we and where are we going?

As the last speaker in this session, Dr. Sommer presented the current pathology state and the recent developments in biomarkers and digitalization in PCa. Dr. Sommer started his presentation by reminding the audience that multiple methods can be used to measure most biomarkers, including immunohistochemistry, fragment length analysis, and sequencing. However, the more complex the method, the higher the cost and turnaround time, which is not always feasible in clinical reality. Therefore, in addition to being highly specific and sensitive, biomarkers should be time- and cost-effective and applicable across a large population. In this context, he questioned the PARP testing strategy currently used in PCa in Germany. As BRCA status testing is costly and takes up to 10 working days, he highly recommended combination treatment strategies, as suggested in the PROpel study. As treatment response can be more easily monitored by PSA change, he suggested that a trial-and-error method might be more feasible than BRCA testing.

In addition to BRCA testing, Dr. Sommer discussed the diagnostic and prognostic value of the phosphatase and tensin homolog (PTEN) in PCa. PTEN is mutated in up to 60% of PCa cases, and complete loss is linked to metastasis and the development of CRPC [72]. PTEN can be detected using immunohistochemistry and in situ hybridization, but whether it is a reliable biomarker is debatable. By presenting the data of a multicenter study, it could be shown that depending on the center, between 12.5 and 51.2 of identical TMA cases were diagnosed with a loss of PTEN [73]. Therefore, the variability in the method and evaluation must still be brought to a higher level, as it was the case with PD-L1 evaluation. Dr. Sommer sees one opportunity in digital pathology: it decreases variability by eliminating the human factor. Moreover, he sees an opportunity to handle multiple complex markers in a timely and cost-efficient manner. However, until now, virtual microscopy and AI-based diagnostics have not been feasible due to high costs, ethics, and user skepticism. However, he is convinced that AI in pathology is inevitable.

### Panel discussion: is ADT still the golden standard in PCa, or have we reached the end of the line?

Based on Dr. Charles Brenton Higgins’s breakthroughs concerning the hormonal treatment of PCa, targeting the AR using ADT or anti-androgens has become the gold standard in PCa treatment [25]. Almost 80 years later, scientists worldwide are still investigating new methods for ADT and developing novel compounds to target AR. With the introduction of the latest generation anti-androgens, apalutamide, enzalutamide, and darolutamide, as well as the CYP17 inhibitor abiraterone, the therapeutic landscape of PCa has been revolutionized, and new therapeutic strategies have become available [74]. These novel compounds showed advantages in single and combination treatments compared with first-generation compounds. Derived from the the Systematic Review and Network Meta-Analysis of Mulati and colleagues, all novel compounds seem to have negligible differences in efficiency alone [75]. However, they showed greater potential for combination treatment [32–36]. Based on this background, Dr. Erb asked, “Is ADT still the gold standard in PCa, or have we reached the end of the line?“. The audience agreed that targeting the AR remains the gold standard for PCa treatment. However, although modern combination therapies increase OS benefits by up to 39%, many patients still develop therapy resistance, and triple therapy does not seem to be the next step [32–36]. Therefore, there is an urgent need for novel therapeutic strategies. One suggested opportunity is to target known PCa features, such as the hyperactive AKT pathway in advanced PCa. AKT is regulated by the tumor suppressor PTEN, a protein that is frequently lost in PCa [72, 76]. Therefore, several trials targeting the PTEN downstream target AKT have been performed [77]. Analysis of the phase III IPATential150 trial revealed that the AKT inhibitor ipatasertib plus abiraterone significantly improved progression-free survival in patients with PTEN-negative mCRPC [78]. However, after the primary progression-free survival readout, the trial was stopped, as there was no significant improvement in OS [79].

In addition to targeting known features of PCa, the panel also discussed targeting known AR features to improve therapy outcomes. Prof. Claessens revealed that targeting the LBD dimerization reduced AR transactivation and PCa proliferation [52]. Next to dimerization, scientists have tried for years to target the N-terminal domain of AR. The first clinical trial showed that the combination of N-terminal AR inhibitor EPI-7386 and enzalutamide is safe and well tolerated and shows durable PSA reduction in 5/6 evaluable patients [80]. The audience agreed that the outcome of the currently running trial (NCT050755779) may be groundbreaking for AR-targeted therapy.

The AR influences several processes in PCa cells [19–22]. Therefore, it was also discussed if targeting pathways altered during ADT or anti-androgen therapy may be a possible therapeutic target. It was therefore assumed that targeting the GR, metabolism, or DNA repair pathways may be ways to improve therapy outcomes.

Finally, it was discussed if the current therapeutic strategies targeted the entire tumor cell population. Prof. Dubrovska already discussed that some PCa stem cell populations are *de novo* resistant to the current therapeutic strategies [27, 56–58]. Furthermore, the current therapeutic approach, including AR-deprivation therapy and RT, might induce tumor cell plasticity [59, 81, 82]. In particular, the development of highly aggressive neuroendocrine PCa may be promoted by targeting the AR signaling pathway [83, 84]. Therefore, the panel agreed that further studies should focus on deciphering the biology of PCa to identify and target heterogeneous PCa stem cell populations and induced tumor cell plasticity.

A final comment mentioned that the development of effective therapies would be hampered without monitoring biomarkers that detect emerging resistance at an early stage. Therefore, multicentre programs such as the prostate biomarkers ProBio trial (www.probiotrial.org30/07/2023) is the first step in the right direction and should be an example to monitor future trials.

## Conclusion

At the symposium, clinicians and scientists exchanged the latest AR research findings to establish collaboration to improve patient care. International participants from Germany, England, the Netherlands, Belgium, Austria, and the Czech Republic discussed various aspects of AR research, and new cooperation could be established. As a result of the favorable reactions from the participants, the organizers hope that the symposium can be held annually in the future and that other non-PCa areas dealing with AR can be included.

## Data Availability

Not applicable.

## Abbreviations

AR: Androgen receptor
AR-V: Androgen receptor splice variants
ADT: Androgen deprivation therapy
AI: Artificial intelligence
CSC: Cancer stem cells
GR: Glucocorticoid receptor
HRR: Homologous recombination repair
LBD: Ligand-binding domain
MAO-A: Monoamine oxidase A
mCRPC: Metastatic castration-resistant prostate cancer
mHSPC: Metastasized hormone-sensitive prostate cancer
MRI: Magnetic resonance imaging
OS: Overall survival
PCa: Prostate cancer
PTEN: Phosphatase and tensin homolog
RT: Radiotherapy
